# Perfluorochemical (PFC) liquid enhances recombinant adenovirus vector-mediated viral interleukin-10 (AdvIL-10) expression in rodent lung

**DOI:** 10.1186/1476-9255-4-9

**Published:** 2007-05-01

**Authors:** John T Li, Laura L Bonneau, Jerry J Zimmerman, Daniel J Weiss

**Affiliations:** 1 University of California, San Francisco, Moffitt M-680, 505 Parnassus Ave., San Francisco, CA 94143, USA; 2 Seattle Children's Hospital & Regional Medical Center, B-9524 Critical Care, 4800 Sand Point Way NE, Seattle, WA, 98105, USA; 3 University of Vermont, room 226C, HSRF, 149 Beaumont Ave., Burlington, VT, 05405, USA; 4 University of Wisconsin School of Medicine, Health Sciences, 750 Highland Ave., Madison, WI, 53705, USA

## Abstract

Adenovirus and cationic liposome mediated transfer of Interleukin-10 (IL-10), a potent anti-inflammatory cytokine, has been shown to decrease pro-inflammatory cytokine levels and overall lung inflammation in models of lung transplantation and injury. Limitations to current approaches of IL-10 gene therapy include poor vector delivery methods and pro-inflammatory properties of human IL-10 under certain conditions. We hypothesize that using perfluorochemical (PFC) liquid to deliver the highly homologous viral IL-10 (vIL-10), which is predominantly anti-inflammatory with minimal pro-inflammatory activities, can potentially be a more effective strategy to combat inflammatory lung diseases. In this study, we compare the use of PFC liquid versus aerosolized method to deliver adenovirus encoding the vIL-10 gene (AdvIL-10) in C57Bl6 mice. Detectable vIL-10 levels were measured from bronchoalveolar lavage fluid and lung homogenates at one, four, ten and thirty days after AdvIL-10. Furthermore, we determined if use of PFC liquid could allow for the use of a lower dose of AdvIL-10 by comparing the levels of detectable vIL-10 at different doses of AdvIL-10 delivered +/- PFC liquid. Results showed that PFC liquid enhanced detectable vIL-10 by up to ten fold and that PFC liquid allowed the use of ten-fold less vector. PFC liquid increased detectable vIL-10 in lung homogenates at all time points; however, the increase in detectable vIL-10 in BAL fluid peaked at four days and was no longer evident by thirty days after intratracheal instillation. In summary, this is the first report utilizing PFC liquid to enhance the delivery of a potentially therapeutic molecule, vIL-10. We believe this strategy can be used to perform future studies on the use of the predominantly anti-inflammatory vIL-10 to treat inflammatory lung diseases.

## Background

Interleukin-10 (IL-10) is a predominantly anti-inflammatory cytokine secreted by activated macrophages, lymphocytes and other cells involved in regulation of the immune response. Some of the known pathways regulated by IL-10 include modulation of Jak/STAT signaling, suppression of T_H_1 T-cell differentiation, and inhibition of macrophage production of pro-inflammatory cytokines [1-6]. Since its discovery over a decade ago, many studies have investigated the potential use of IL-10 to treat inflammatory diseases ranging from arthritis to sepsis. One area of research that shows promise is the use of gene transfer-mediated IL-10 expression in the treatment of lung inflammatory diseases. IL-10 gene therapy has been successful at preserving lung integrity and curtailing an over-exuberant inflammatory response in murine lung pneumonia and systemic inflammation models [7-10]. Furthermore, inflammatory and immune-mediated lung injury, including obliterative bronchiolitis, remains a major cause of morbidity and mortality for lung transplant recipients. Recombinant adenovirus vector mediated IL-10 expression in lung epithelium was successful at reducing obliterative bronchiolitis in an experimental heterotopic rat lung transplant model[11].

However, both human and murine IL-10 can also have pro-inflammatory action under certain conditions. In contrast, the highly homologous viral IL-10 derived from Epstein-Barr virus BCRF-1 protein is predominantly anti-inflammatory with minimal pro-inflammatory activities; therefore, vIL-10 is potentially a better candidate than human IL-10 (hIL-10) for treating inflammatory diseases [12-14]. For this reason, our studies were conducted with vIL-10 instead of human or mouse IL-10.

Feasibility of IL-10 gene therapy for lung diseases hinges on developing an effective delivery system and likely compartmental expression restricted to the lung. Due to the short half-life of IL-10, strategies utilizing recombinant IL-10 protein require many repeated doses and makes long-term administration impractical. To circumvent this problem, gene therapy strategies remain appealing. Previous studies have used lipid carriers to enhance plasmid mediated IL-10 delivery with limited success[10]. Many other groups have reported using viral vectors to deliver IL-10 with greater success, but still likely have not identified the most effective method of delivery. Furthermore, these studies often used large amounts of adenovirus vectors raising concerns for adenovirus-associated toxicity. In response to these concerns, recent studies using other recombinant vectors to deliver IL-10 to lung epithelium have been reported. For example, recombinant sendai virus has been used to successfully improve IL-10 gene delivery to treat a murine model of bronchial fibrous obliterans after tracheal transplantation[15]. Developing a strategy to boost IL-10 delivery to the lung may be beneficial in future experimental lung injury research and successful lung gene therapy in humans.

We have developed a method to enhance adenovirus delivery by utilizing perfluorochemical (PFC) liquid[16]. PFC liquids have been safely used in liquid ventilation trials in humans and various experimental animal models [17-24]. We have used PFC liquids to improve adenovirus and adeno-associated virus delivery in rodents and non-human primates[16,25,26]. Previously, our studies have been limited to delivery of the reporter gene beta-galactosidase. With the current investigation, we sought to determine the effectiveness of utilizing PFC liquid to deliver a potentially beneficial biologically active gene, vIL-10, in AdvIL-10.

## Methods

### Adenovirus viral IL-10 construction

Recombinant type 2 E1a/E1b deleted, replication deficient, adenovirus carrying the viral IL-10 gene driven by the CMV promoter was kindly provided by Dr. Jay Kolls (Louisiana State University). This construct is designated as AdvIL-10 for this report.

### AdvIL-10 transduction of lung epithelial cells

A549 transformed human lung epithelial cells (American Type Culture Collection^®^, Manassas, VA) were cultured in DMEM media containing fetal bovine serum 10% (HyClone^®^, Logan, UT), L-glutamine 1% (Bio-Whittaker, Walkersville, MD) and Penicillin 2% (Bio-Whittaker, Walkersville, MD). A549 cells were incubated in a humidified 37°C incubator with 21% oxygen and 5% carbon dioxide. Cultures were grown in tissue-culture twelve-well plates (Nunc™) until cells were 70–80% confluent. A multiplicity of infection (MOI) of 1000:1 of AdvIL-10 was used to transduce the A549 cells. Supernatant was collected and stored at -80°C.

### Vector and PFC Liquid Instillation

All animal studies were approved by the IACUC of the Fred Hutchinson Cancer Research Center and conformed to AAALAC and institutional IACUC standards. Male 6–8 week old C57Bl/6 mice (Charles River, Wilmington, MA) were housed and fed ad libitum in accordance to institutional animal use protocols. Mice weighed 19–24 grams at the time of the procedure. The procedure for administration of vector and PFC liquid by transtracheal puncture has been described in detail^16^. In brief, mice were anesthetized with an intraperitoneal injection of Avertin (tribromoethanol and tertiary amyl alcohol) 1 mg/kg and immobilized in supine position with head elevated 30°. Supplemental 100% oxygen was given at 5–10 liters per minute to the nares for the duration of the procedure. The skin was prepped with 70% ethanol and the trachea was exposed by cut-down with a scalpel and blunt dissection. The strap muscles overlying the trachea were carefully spread to expose the trachea. A 0.5 mL 27 gauge insulin syringe (Becton-Dickinson, Franklin Lakes, NJ) was used to deliver 50 μL of either AdvIL-10 or Adlac-z at the desired concentration by puncturing the trachea just below the larynx and instilling the vector over 1–2 minutes. PFC liquid (Perfluoro-chemical FC-75^®^, ACROS Organics, Fisher Scientific, Fairlawn, NJ) was pre-oxygenated by bubbling 100% oxygen through a 23 gauge needle submerged in 5–10 mL of PFC liquid for 2 minutes. Immediately following vector administration, PFC liquid was instilled with a 1 ml syringe and 25 gauge needle at a dose of 10 mL/kg body weight (190 μL to 240 μL) through the same puncture site used the deliver the adenovirus vector. Aerosolized vector delivery was performed by delivering 10 mL/kg body weight of air following vector instillation. The incision was sutured closed with 2-0 silk in an interrupted fashion. Mice were recovered under a heat lamp in an oxygenated cage until fully awake and active. Mice were observed daily for illness. Overall mortality rate was 8%.

### Lung Harvest and Analyses

#### Bronchoalveolar Lavage

At the designated times after vector administration, treated mice (n = 3–5) along with naïve control mice (n = 3) were euthanized with an over an overdose of Avertin and immobilized in a supine position. The heart-lung block was exposed by cut-down technique and a blunted 23-gauge butterfly needle inserted into the trachea and secured with 2-0 silk ligature. The lungs were lavaged with 1 mL PBS containing 0.6 mM EDTA and protease inhibitor cocktail (PMSF 1 mM, leupeptin 1 μg/mL, aprotinin 2 ng/mL) using a 3 mL syringe. The lavage was repeated one time. Total cell counts were obtained using a hemacytometer and cell differentials determined on cytospin preparations by DiffQuick™ (International Reagent Corp., Kobe, Japan) staining. Total protein levels were measured with the BCA protein assay (Pierce, Rockford, IL).

#### Determination of vIL-10 content in BAL fluid and lung homogenates

After performing BAL, the right lower lobe was removed and snap frozen in liquid nitrogen and stored at -80°C. The right lower lobe was homogenized in 1 cc sterile filtered lysis buffer (NaCl, KCl, Triton-X-100 2%, Tris-HCl 1 mM, PMSF, leupeptin, aprotinin, and pH 7.4). Debris was removed by centrifuging lung homogenate at 13,000 g for 10 minutes at 4°C. The supernatant was decanted and stored at -80°C.

### IL-10 ELISA

IL-10 levels in the BAL fluid and lung homogenate were measured with a human IL-10 ELISA kit specific for human and viral IL-10 protein according to manufacturer's instructions (Immunotech, Marseille, France). Importantly, the IL-10 ELISA kit did not detect mouse IL-10.

### Statistics

Results of IL-10 ELISA and cell counts were analyzed using unpaired student t-test. Statistical significance was considered as a p-value less than 0.05.

## Results

### AdvIL-10 Mediated Viral IL-10 Expression In A549 Lung Epithelial Cells

**Figure 1:**
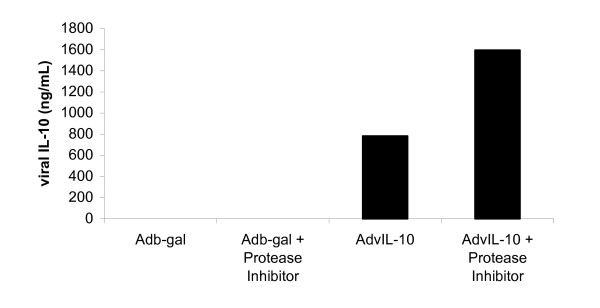
**AdvIL-10 infects lung A549 epithelial cells to produce detectable vIL-10 protein in the culture media and addition of protease inhibitor cocktail preserves detectable vIL-10**. A549 cells at were infected with 1000:1 MOI of either Ad*lac-*Z or AdvIL-10. Supernatants collected +/- protease inhibitor cocktail and assayed for vIL-10 by ELISA. Values represent average of two experiments.

Viral IL-10 levels were measured from cell culture media after AdvIL-10 administration at 1000:1 MOI. Viral IL-10 was readily detectable by ELISA. The detectable levels of viral IL-10 was nearly two times greater when the culture medium was treated with protease inhibitor cocktail (1,600 ng/ml) compared to culture media collected without protease inhibitor cocktail (780 ng/ml) (Fig 1). Hence, the remaining experiments were performed in the presence of protease inhibitor cocktail in the collection medium.

### PFC Liquid Increases Detectable IL-10

**Figure 2:**
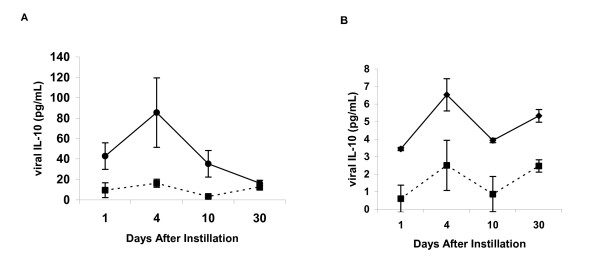
**Use of PFC liquid increases detectable viral IL-10 in BAL fluid (A) and lung homogenate (B)**. Viral IL-10 levels from BAL fluid or right lower lobe lung homogenate were collected at 1, 4, 10 and 30 days after intratracheal instillation of AdvIL-10 with PFC liquid (■) or AdvIL-10 with air (●). Each time point represents the mean ± SEM (* p-value < 0.05). Representative experiments from repeated experiments (n = 3–5/group).

To determine the effect of PFC liquid on AdvIL-10 delivery, C57 mice were instilled with 1 × 10^9 ^particles of AdvIL-10 with and without PFC liquid. Viral IL-10 levels were measured in BAL fluid at one, four, ten and thirty days after intratracheal instillation, and the levels were compared to reflect the level of viral IL-10 transgene expression. The ELISA kit used to measure viral IL-10 levels was specific for viral IL-10 or human IL-10 and did not detect endogenous murine IL-10. Viral IL-10 levels were significantly higher in the mice receiving AdvIL-10 with PFC liquid versus AdvIL-10 with air (Fig. 2a). The largest difference in viral IL-10 levels, a five-fold increase, was detected in BAL fluid collected four days after intratracheal AdvIL-10 delivery. The difference was no longer appreciated at thirty days. In contrast, vIL-10 levels measured from right lower lobe lung homogenates in the AdvIL-10 + PFC group were approximately two-fold higher than the AdvIL-10 + air group at each time point (Fig. 2b). Although the overall boost in detectable vIL-10 was lower when measured in lung homogenates compared to BAL fluid measurements, the increase in vIL-10 levels was statistically significant at all time points and persisted to the longest measured time point of thirty days.

### PFC Liquid Permits A Lower Dose Of AdvIL-10

**Figure 3:**
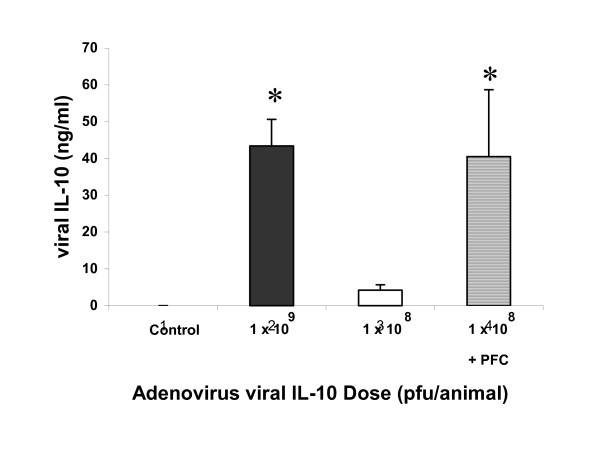
**PFC liquid allows for use of a lower dose of AdvIL-10**. BAL fluid viral IL-10 levels fluid from mice treated with lower dose 1 × 10^8 ^particles of AdvIL-10 with PFC liquid (≡), lower dose 1 × 10^8 ^particles AdvIL-10 with air (□), or higher dose 1 × 10^9 ^particles AdvIL-10 with air (■). Control animals did not receive AdvIL-10. Values represent mean ± SEM (* p-value < 0.05 when compared with lower dose 1 × 10^8 ^particles AdvIL-10 alone group, n = 4 per group).

We sought to determine if PFC liquid could allow the use of a lower dose of AdvIL-10. We delivered a ten-fold lower dose of AdvIL-10, 1 × 10^8 ^particles per animal, and compared the detectable IL-10 levels obtained with an AdvIL10 dose of 1 × 10^9 ^particles per animal. Use of PFC liquid to deliver a ten-times lower dose of AdvIL-10 (1 × 10^8 ^particles/animal) showed similar detectable IL-10 levels in the BAL fluid when compared to a ten-fold higher dose of AdvIL-10 (1 × 10^9 ^particles/animal) instilled without PFC liquid when measured at four days after vector instillation, the time of peak vIL-10 expression (Fig. 3).

### PFC Liquid Did Not Increase Lung Inflammation Provoked by the Adenovirus Vector

**Figure 4:**
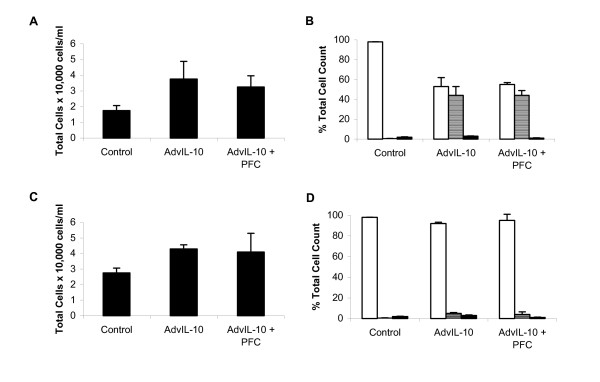
**Use of PFC liquid does not potentiate transient lung inflammation provoked by adenovirus delivery**. (A) Total cell count from BAL fluid collected 1 day after intratracheal instillation of AdvIL-10 with PFC liquid or AdvIL-10 with air. (B) Cell differential (□ %macrophage, ≡ %neutrophil, ■ %lymphocyte) from BAL fluid collected at 1 day after intratracheal delivery of AdvIL-10 with PFC liquid or AdvIL-10 with air. (C) Total cell count from BAL fluid collected 4 days after intratracheal instillation of AdvIL-10 with PFC liquid or AdvIL-10 with air. (D) Cell differential (□ %macrophage, ≡ %neutrophil, ■ %lymphocyte) from BAL fluid collected at 4 days after intratracheal delivery of AdvIL-10 with PFC liquid or AdvIL-10 with air. Control animals did not receive adenovirus instillation. Values represent mean ± SEM.

Previous experience with PFC liquid in animal models demonstrated a pro-inflammatory effect of PFC liquid in the lung. We measured the cell count and cell differential from the BAL fluid collected at one and four days after PFC liquid administration (Fig. 4). Total cell counts from BAL fluid collected one day after mice received intratracheal instillation of AdvIL-10 with PFC liquid (375,000 cells/mL) and AdvIL-10 with air (325,000 cells/mL) were not statistically different. Both values were statistically higher than the control total cell count of (275,000 cells/ml). Similar results were obtained from BAL fluid total cell counts obtained four days after intratracheal instillation. The cell differential one day after vector +/- PFC liquid instillation showed a significant increase in neutrophil count in both the AdvIL-10 + PFC liquid group and the AdvIL-10 group (53% macraphages, 44% neutrophils, 3% lymphocytes versus 55% macrophages, 44% neutrophils, 1% lymphocytes, respectively) as compared to the naïve control animals (98% macrophages, 0.4% neutrophils, 2% lymphocytes). By day four the cell differential in both AdvIL-10 groups returned to baseline values with a prdominance of marcrophages greater than 92% and neutrophils less than 6%.

## Discussion

In this study, we have demonstrated that use of PFC liquid enhances intratracheal delivery of recombinant AdvIL-10 to rodent lung. Use of PFC liquid increased the amount of detectable vIL-10 by up to five fold. Furthermore, PFC liquid decreased AdvIL-10 dose by ten-fold to achieve similar results as AdvIL-10 delivered with air. This is the first report demonstrating the feasibility of delivering a potentially biologically therapeutic gene utilizing PFC liquid in animals.

We demonstrated a disparity in the kinetics of measurable vIL-10 from BAL fluid and lung homogenate samples. PFC liquid increased detectable vIL-10 levels from BAL fluid, but the boost from PFC liquid diminished over time, eventually showing no difference from the non-PFC group by day thirty. In contrast, vIL-10 levels measured from lung homogenates showed a steady difference in detectable vIL10 at all time points, extending to the longest measured time points of thirty days. This data suggests that detectable vIL-10 levels vary depending on the compartment that it is measured. Our findings are consistent with prior reports indicating that vIL-10 transgene expression can be measured for an extended period time in lung tissue, with the longest reported time period extending beyond 42 days after intratracheal instillation[7,27]. The diminished detectable secreted vIL-10 in the BAL fluid despite increased intracellular vIL-10, suggests that over time, secreted vIL-10 is either degraded more rapidly in the extracellular environment or the secretion of vIL-10 is decreased. Rather than increased systemic expression, localized over expression of vIL-10 may in fact be more beneficial. In murine models of sepsis, compartmental delivery of IL-10 has been shown to be beneficial, whereas systemic delivery showed no protection from lethality[28]. Moreover, other IL-10 studies in murine sepsis suggest systemic over-expression of IL-10 may be deleterious due to its potential to create a state of immune paralysis by producing a TH2 state and suppressing TH1 cytokine production[29,30]. Therefore, developing an efficient method for over-expressing IL-10 localized to the lung could prove to be critical in a successful strategy to combat lung inflammatory diseases such as ARDS and transplant rejection.

The mechanism of how PFC liquid improves transgene expression is still unclear. The first assumption is that PFC liquid acts as a carrier to allow for improved distribution of adenovirus throughout the lung. Although we believe this may be true, PFC liquid appears to have more activity than a mere inert carrier. Previously, we have demonstrated that PFC liquid transiently opens tight junctions, which in turn, may allow adenovirus to gain better access to its receptors on the basolateral surface of epithelial cells[31]. Furthermore, nebulized PFC liquid up to one hour prior to adenovirus delivery appears to enhance transgene expression (unpublished data from our laboratory).

The use of PFC liquid allows for an increase in detectable IL-10 and can be potentially beneficial for applications of curbing lung inflammation. One area of particular interest is to use PFC liquid to deliver AdvIL-10 to the donor lung to improve lung function and graft survival. Several animal models have successfully demonstrated the potential benefits of pre-conditioning the donor lung to over-express IL-10 [32-35]. We hope the use of PFC liquid will aid future studies aimed at evaluating the potential use of vIL-10 in treating inflammatory lung disease.
